# Colonoscopy procedure simulation: virtual reality training based on a real time computational approach

**DOI:** 10.1186/s12938-018-0433-4

**Published:** 2018-01-25

**Authors:** Tingxi Wen, David Medveczky, Jackie Wu, Jianhuang Wu

**Affiliations:** 1 Software School, Xiamen University, Xiamen, Fujian China; 2 School of Medicine, Western Sydney University, Campbelltown, NSW Australia; 3 Shenzhen Institutes of Advanced Technology, Chinese Academy of Sciences, 1068 Xueyuan Boulevard, Xili Nanshan, Shenzhen, 518055 China

**Keywords:** Colonoscopy, Simulator, Virtual reality, Elastic rod model, Interactive training

## Abstract

**Background:**

Colonoscopy plays an important role in the clinical screening and management of colorectal cancer. The traditional ‘see one, do one, teach one’ training style for such invasive procedure is resource intensive and ineffective. Given that colonoscopy is difficult, and time-consuming to master, the use of virtual reality simulators to train gastroenterologists in colonoscopy operations offers a promising alternative.

**Methods:**

In this paper, a realistic and real-time interactive simulator for training colonoscopy procedure is presented, which can even include polypectomy simulation. Our approach models the colonoscopy as thick flexible elastic rods with different resolutions which are dynamically adaptive to the curvature of the colon. More material characteristics of this deformable material are integrated into our discrete model to realistically simulate the behavior of the colonoscope.

**Conclusion:**

We present a simulator for training colonoscopy procedure. In addition, we propose a set of key aspects of our simulator that give fast, high fidelity feedback to trainees. We also conducted an initial validation of this colonoscopic simulator to determine its clinical utility and efficacy.

## Background

Colorectal cancer (CRC) ranks as one of the most prevalent and significant causes of morbidity and mortality in the developed world [1]. This disease mostly develops from adenomatous polyps [2], which develop into CRCs. If their progress is unchecked, CRCs can aggressively penetrate the colonic wall and metastasize to vulnerable locations such as the liver or lungs through vascular and lymphatic spread [3]. As a result, regular screening, early diagnosis and effective treatment is paramount to decrease the mortality and morbidity caused by this disease. Lower gastrointestinal endoscopy (i.e. colonoscopy and sigmoidoscopy) is a key player in the management of patients at risk of CRC. It is used as screening technique alongside faecal occult blood testing (FOBT), double contrast barium enema and computer tomographic colonoscopy (CTC) (among other tests) [4]. It can also diagnose malignancy or pre-malignant adenomatous polyps through biopsy and polypectomy of suspicious polypoid masses. It can also prophylactically treat CRC through polypectomy and periodic surveillance [3].

It has been suggested that primary care physicians perform colonoscopies in order to better manage the increasingly high burden of disease caused by CRC [5]. It seems reasonably foreseeable that the demand for colonoscopy training will increase not only there, but throughout the developed world. It is therefore desirable and expedient that physicians become proficient in colonoscopy as quickly and as easily as possible. We present details of the colonoscopic procedures, how gastroenterologists are trained and our proposed simulation training techniques as follows.

### Colonoscopy procedure

In colonoscopy, a scope measuring approximately 7–9 mm in diameter is inserted into the patient’s rectum and advanced into the colon up until the caecum/terminal ileum. The endoscopist can either sit or stand while using pushing, pulling, torsion, hooking and sliding movements in order to advance the colonoscope throughout the colon [5]. The colonic mucosa is constantly examined through the use of a camera on the distal tip of the colonoscope. In the case that the view is obstructed, the endoscopist can use suction or air insufflation to clear the view [5]. In the case that the endoscopist encounters a polyp, the endoscopist can biopsy it and use forceps/snare to excise it from the mucosa (polypectomy).

### Change in teaching paradigms

There has been a movement away from the traditional ‘see one, do one, teach one’ model to the use of virtual simulators to train colonoscopy. This is for several reasons: (1) colonoscopy is a difficult and time consuming procedure to master, with previous studies suggesting that up to 700 performed procedures are required to gain proficiency [6]; (2) it is ethically questionable to train colonoscopy on real patients, as a poorly performed colonoscopy can have very serious complications including perforation, bacteriaemia and haemorrhage [7]; (3) it is an expensive model as the productivity of qualified endoscopist suffers while supervising trainees compared with performing procedures independently. It should be noted that virtual simulators are expensive and are only a valuable investment if proven to be valid and high fidelity [8]; (4) the traditional model does not offer an objective standard of success in achieving proficiency in colonoscopy. In a clinical environment where the colonoscopist’s skill and style can have wide-ranging variations that influence the quality and safety of their colonoscopy procedures; [9] colonoscopy simulators can aid in providing objective parameters that can standardise optimum techniques and strategies; (5) the skills gained by using colonoscopy simulators seem to be transferable to real life patient situations [10] and retainable for at least 4 months [11]. Furthermore, an attempt has been made to establish competency criteria for training using simulators [12]. Due to the above reasons, it is reasonable to encourage this paradigm shift towards simulation environments for training novices where possible.

### State of virtual simulators

Virtual simulators provide safe, realistic environments for trainees to learn the skill of colonoscopy. Trainees can understand 3D relationships between anatomical structures in the colon and develop the haptic control and finesse required to operate the colonoscope. Intraoperative events such as perforation or haemorrhage can be managed in a safe and low-stress environment without putting a patient at risk. Furthermore, the progress of trainees can be objectively measured by several parameters including percentage of the mucosa visualised, time taken for caecal intubation, level of patient discomfort and number of intraoperative events/errors. Simulators may also offer the opportunity for experienced endoscopists to rehearse colonoscopy procedures with patient-specific data in order to foresee any potential challenges during the real colonoscopy.

Several virtual reality colonoscopy simulators and diverse haptic interfaces have been proposed and developed. Yi et al. proposed a simulator whose haptic interface allowed for the ‘jiggling’ motion in colonoscopy to straighten the colonoscope and shorten the bowel [13]. De Visser et al. incorporated loop formation into their simulator by modelling tissues surrounding the colon [14]. Samur et al. instrumented a real clinical colonoscope in their haptic interface for improved haptic fidelity [15]. US company Simbionix offers the commercial product GI Mentor 2 which includes features like the scope indicator pain indicator and simulates cases based on real patient data [16]. The CAE EndoVR (previously Accutouch) allows trainees to perform simulation of polypectomy, biopsy and haemostasis [16]. Olympus offers the Endo TS-1 commercial colonoscopy simulator which provides a real time simulation for trainees [16]. King et al. recently developed a low-cost physical simulator with the aim to be more accessible and affordable for residency programs [17]. Wong et al. also mentioned other development of biomedical devices [18].

### Our approach

In previous work, we have completed some simulation research for percutaneous coronary intervention and the behavior of guide-wire in vascular interventional radiology [19, 20]. The evaluating the array of colonoscopy simulators available, we identified some key aspects that have been developed that play significant roles in a high fidelity and realistic colonoscopy simulator. It is different from most previous methods [21–23].

1. Model surrounding organs to allow loop formation and simulate the dynamic nature of the bowel.
2. For haptic interface, an actual colonoscopy should be used to allow better haptic fidelity and better likeness to real life colonoscopy.
3. Create cases and histories that vary in difficulty and also have randomly generated cases to prevent trainees from ‘gaming’ the simulator.
4. Includes translational motion that shortens colon and also yaw and pitch movements to the tip of the colonoscope for better haptic fidelity.
5. Integrate procedures such as biopsy, haemostasis and polypectomy into the simulation environment as they are necessary for proficiency in colonoscopy.

In this paper, we will present a real-time, valid simulation environment for colonoscopy training. It contains an excellent surface modelling for the colonic anatomy. The colonoscope is represented by a physics-based elastic rod model which discretize into different resolutions that dynamically adapt to the diameter and curvature of the colon. To dramatically reduce computational effort and time, we propose a force correction strategy that adjusts the elastic force between the colonoscope and bowel wall after collision detection. We also allow user-induced perforations so that trainees can manage intra-operative complications in a safe and low-stress environment.

The rest of this paper is presented as follows: details of our simulator are described in “Methods” section, followed by experimental results and discussion of the physical rod model and virtual simulator in “Results” section. Finally, “Discussion” section contains the conclusion and comments on possible improvements in future works on the topic of colonoscopy.

## Methods

In this section, we firstly outline the structural overview of our proposed colonoscopy simulator. Secondly, we introduce our geometric modelling method for the colon and surrounding structures to give rise to a realistic simulation environment. Thirdly, we describe our colonoscope-colon physic-based system. Finally, we briefly describe the haptic interface and hardware of the simulator.

### System overview

**Fig. 1:**
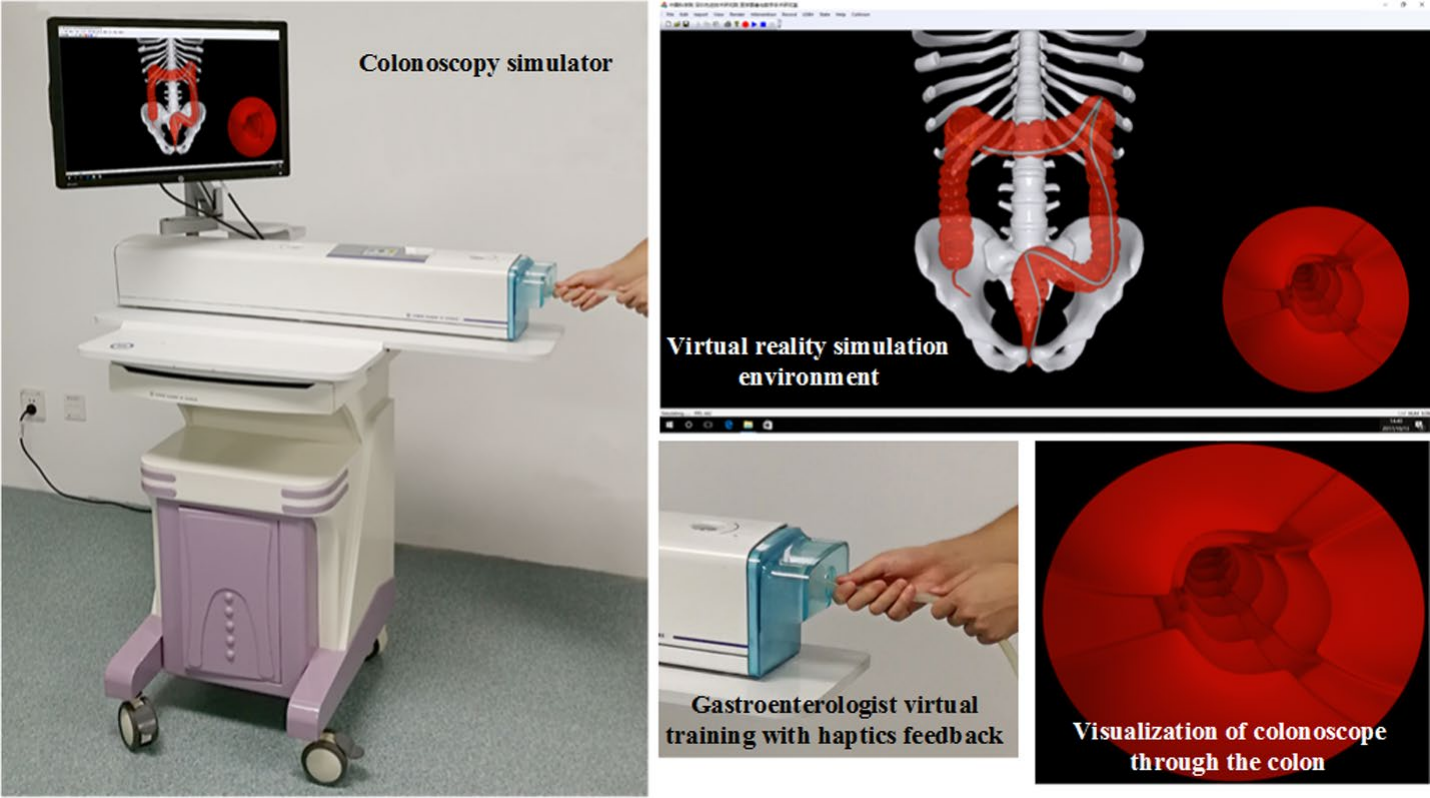
Simulation system depicting use of the colonoscope by a gastroenterologist trainee

**Fig. 2:**
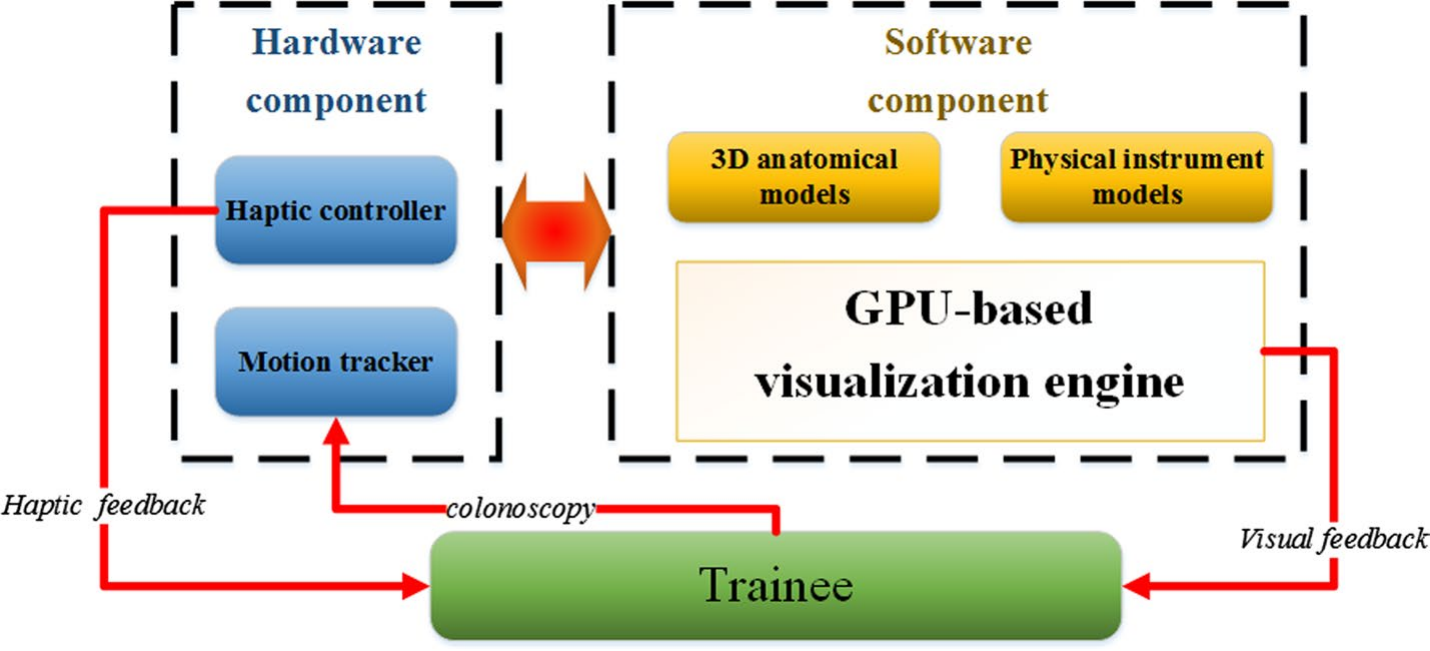
Architecture of colonic simulation environment

Our simulator offers both realistic visual and haptic feedback to endeavor to produce natural and life-like interactions between the trainee and simulation environment. The relationships between the different components of our simulator including the endoscopic view and the trainee are presented in Fig. 1. The colon simulator provide a virtual reality simulation environment show the realistic visual feedback similar to what an operator could see in interventional operations and a haptic apparatus that can interact with trainees naturally, just like the trainees are in the real interventional procedures. The architecture of our simulation system and the trainees are shown in Fig. 2. The system includes hardware component and software component. The former component consist of a haptic controller and a motion tracker; the latter component consist of 3D anatomical models, physical instrument models and GPU-based visualization engine. The motion tracker can track trainees’ behavior, and the 3D anatomical models and physical instrument models process, then the haptic controller and GPU-based visualization engine offer haptic feedback and visual feedback, respectively.

### Geometric modeling

**Fig. 3:**
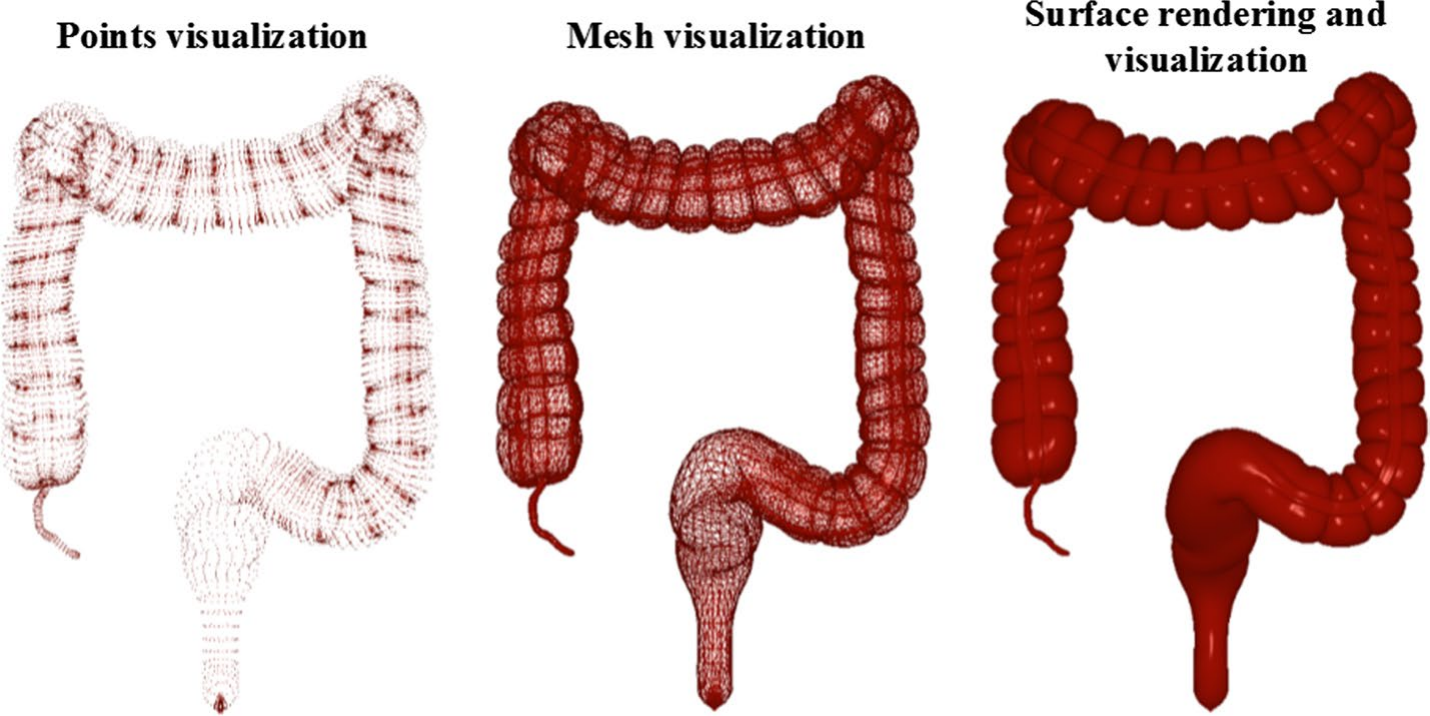
Geometric modeling of colon and meshing of model

Accurate anatomical modelling is the foundation of a high-fidelity simulator. The technique of modelling is varied with some using computer aided design tools or marching cubes to achieve surface modelling. However, we have used a scale-adaptive surface modelling approach for the following reasons: (1) the less amount of triangles reduced the calculation of rendering and collision detection. (2) Less aliasing artifacts and higher quality surface create a more realistic anatomical model. (i) The 3D model is inputted and extracted using adaptive up-sampling in this stage, then a 3D point set with high densities will be outputted, as shown in Fig. 3 (left). (ii) The second stage include two sub-stages. Sub-stage one is that the normal vector of each point is calculation via covariance analysis of the points obtained by *k*-nearest neighbor algorithm; Sub-state two is that all the directions of the vectors is mode via normal propagation based on minimal spanning tree, s illustrated in Fig. 3 (middle). (iii) This stage is to construct indicator function that focus on implicitly describing the underlying 3D point set, which is calculated by solving a Poisson equation constructed from the normal vector field. (iv) The stage also consist of two sub-stage. Sub-stage one is mesh-expanding, an initial mesh composed of six triangles surrounding a given point, whose edge lengths are adaptively determined according to the local curvature radius of the point, is firstly constructed and is subsequently expanded by gradually growing triangles from its boundary edges; sub-stage two is gap-stitching that fill the gaps yielded in the mesh generation. The gap is sewed by triangulating the polygon formed from the boundary edge of the gap.

### Colonoscope modelling

**Fig. 4:**
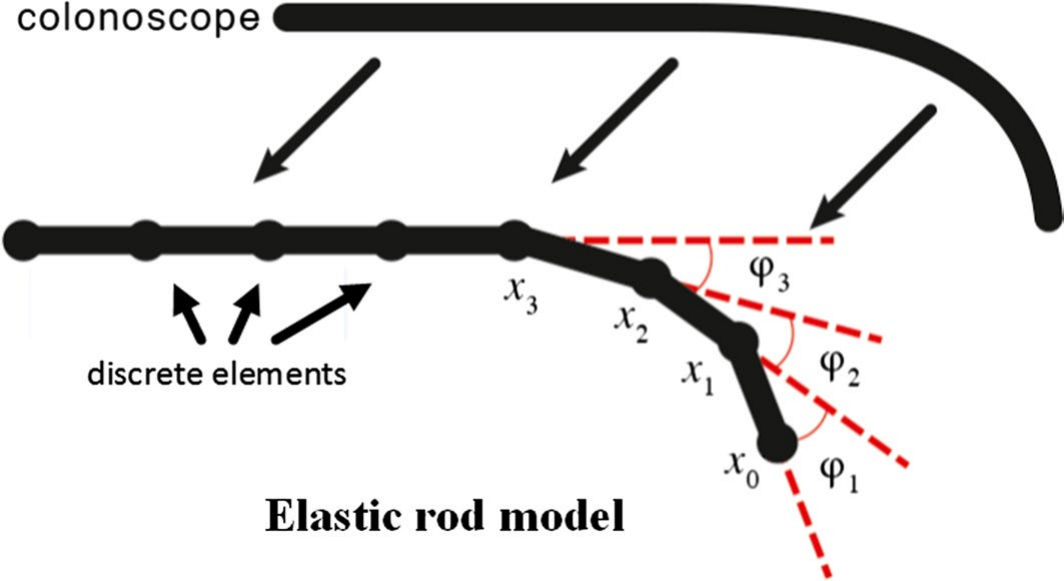
Discrete model representing the flexible colonoscope structure

A dynamic, computationally-efficient approach to modelling the colonoscope is essential in creating a real-time and high fidelity simulation environment. In this work, the colonoscope is represented by a chain of rigid rods, which performs twisting rather than stretching action. The collision detection and force propagation are based on these joint points. As shown in Fig. 4, the model of colonoscope is composed of joint points and rods, and each point links two rods. Let *x*_*i*_ represents *i*th point and a vector *t*_*i*_ represents *i*th rod, which is defined as follows:

1 $$t_{i} \, = \,x_{i - 1} \, - \,x_{i} , \quad i\, \ge \,1.$$

The bending energy of the rod model is measured by the angle *φ*_*i*_ between two adjacent rods *t*_*i*_ and *t*_*i*-1_. To simulate intrinsically curved tips with different curvatures, bias angles (e.g. *φ*_1_, *φ*_2_, *φ*_3_, …,) as shown in Fig. 4. In addition, It is need to be predefined a maximum bias angle *φ*_*m*_ all joint points in order to let the colonoscope bend gradually.

In order to save computational effort and avoid modelling-induced perforation of the bowel wall, we propose an algorithm that dynamically discretizes the rods with regard to the diameter and curvature of the colon. Let *n*_*d*_ denotes the times of discretization. It is calculated as follows:

2 $$n_{d} = \left[ {log\frac{{\bar{l}_{t} }}{{(r_{c} + 0.5d_{v} )\sqrt {2(1 - cos\varphi_{m} )} }}} \right],$$

where *d*_*v*_ denotes the diameter of vessel, $\bar{l}_{t}$ is the average length of the rods, *r*_*c*_ is the radius of curvature and *ψ*_*m*_ is the maximum bias angle of adjacent rods. From the above, it can be seen that *n*_*d*_ changes with the diameter and curvature of the colon. Therefore, when the colonoscope is passed through a section of the colon with high-curvature or small-diameter, *n*_*d*_ is positive and each rod splits into $2^{{n_{d} }}$ smaller ones to avoid perforation and visual artifacts caused by low resolution modeling. However, when colonoscope is passed through low-curvature or large-diameter area, *n*_*d*_ is negative and $1/2^{{n_{d} }}$ rods merge together. This decreases the amount of joint points whose positions need to be calculated, which decreases computational effort (see 2.4 Collision detection). To further decrease computational effort, *n*_*d*_ is calculated in pre-processing stage instead of in real time simulation and corresponding trigger points for splitting/merging are planted in certain area for further discretization/merging.

It can be seen that this method also eliminates artificial perforation of the bowel wall by reducing rod length in small-diameter areas where greater rod lengths could not pass smoothly. However, it is important to note that iatrogenic perforation is a valuable and useful experience for simulation and is preserved in our approach (see Perforation).

### Physical modelling

#### Collision detection

When the colonoscope is acted upon by a push/pull/torsion motion, collision detection is performed to update the position of the colonoscope relative to the bowel wall. As this results in a vast number of detections per simulation, we present an efficient and fast collision detection computation in order to guarantee a real time experience. We use an open-source collision detection library called OPCODE, which uses memory-optimized bounding volume hierarchies to minimize memory occupation without sacrificing efficiency. A bounding sphere is assigned to every point between the rods after updating their coordinates after the movement. The radius of the sphere is half the distance between the points in order to cover the whole of the colonoscope. This ensures that inter-penetration between the rods and the bowel wall is avoided. If a collision is detected, the bowel wall deforms due to the pushing effect of the bounding sphere. According to Hooke’s law, the elastic force generated by this deformation can be expressed as follows:

3 $$F_{bowel} = kd,$$

where *k* is the modulus of elasticity and *d* is the depth of the overlap. The direction of the force is perpendicular to the contact triangle face that has been collided with. If the collision involves more than one triangle, the resulting force equals the sum of all component forces. There are two cases of collision in the detection: the joint point (1) outside or (2) inside the bowel wall. In (1) the bounding sphere as well as the joint point is outside the bowel. The depth of the overlap can therefore be calculated as:

4 $$d = r + s,$$

where *r* is the radius of the bounding sphere and *s* is the minimum distance from the joint point to the colliding triangle face. For case (2) the joint point is inside the vessel and the depth of the overlap is instead calculated as:

5 $$d = r - s.$$

#### Collision response

In our simulation, the colonoscope and the colon can be considered to be a small physical system. If a collision is detected, the elastic force generated by the deformation in the bowel wall acts against the colonoscope, resulting in a complex continual change in position for both the colonoscope and the bowel wall. This process involves a rapid exchange of energy inside the system until equilibrium, or the state whose total potential energy *E*_*total*_ is minimum, is achieved. The system’s total energy is defined as follows:

6 $$E_{total} = E_{c} + E_{b} .$$

where *E*_*c*_ equals the bending energy of the colonoscope and $E_{v}$ equals the elastic energy of the bowel wall. We define *E*_*c*_ as follows:

7 $$E_{c} = \mathop \sum \limits_{i = 2}^{n} \frac{1}{2}C_{i} (\theta_{i} - \varphi_{i} )^{2} .$$

*θ*_*i*_ denotes the angle between adjacent rods of point *x*_*i*_, while *φ*_*i*_ denotes the bias angle of *x*_*i*_ (see Fig. 1). The energy of bowel wall *E*_*b*_, is defined as follows:

8 $$E_{nb} = \mathop \sum \limits_{i = 2}^{n} \frac{1}{2}k_{v} d_{i}^{2} .$$

where *k*_*v*_ denotes the elastic coefficient of the bowel wall and *d*_*i*_ equals the depth of the bowel wall’s deformation. By iteratively calculating the joint points’ displacement until the *E*_*total*_ reaches its minimum, the colonoscope’s position at equilibrium is found. In each iteration, the elastic force applied by the bowel wall changes and needs to be calculated for the subsequent computation. In order to accelerate the convergence of the iteration, we propose our force correction algorithm as follows:

9 $$F_{i}^{'} = F_{i} \left(1 - \eta \alpha_{i} \frac{{F_{i} }}{{\left| {F_{i} } \right|}}\right).$$

where *η* is the feedback coefficient which denotes the scale of the force’s reduction induced by the displacement. *F*_*i*_ is the external force in the previous iteration while *F*_*i*_ is the newly updated force. With this strategy, collision detection only needs to be performed once, which reduces computational burden and allows for an efficient, real-time simulation. Provided that the feedback coefficient is set correctly, the external force is found efficiently and joint points reach their equilibrium in fewer number of iterations.

#### Perforation

Perforation of the bowel wall is a severe consequence of applying excessive force to the colonoscope in colonoscopy and carries various sequelae that can cause serious morbidity and mortality. In order to account for this possibility, we apply a user-defined boundary force *F*_*r*_ to define the rupture strength of the bowel wall. When the system total energy’s reaches minimum and collision response is completed, the elastic force applied by the colonoscope to the bowel wall is calculated and compared with *F*_*r*_. If the elastic force is greater than *F*_*r*_; the bowel wall is perforated.

### Haptic interface

The hardware component involves a haptic controller as well as a motion tracker. The tracker is responsible for measuring the translation (pushing and pulling) and rotation (twisting) of both catheter and guide wire and thus comprises two modules: rotation detection and translation detection. The rotation module includes encoder raster, encoder disc, and dummy revolving hollow rod which is connected to the encoder disc. When manipulating the hollow rod, the rotation will be detected by the encoder grating, and the signal is simultaneously transmitted to the controller to control instrument model rotating in the colonic model.

## Results

**Fig. 5:**
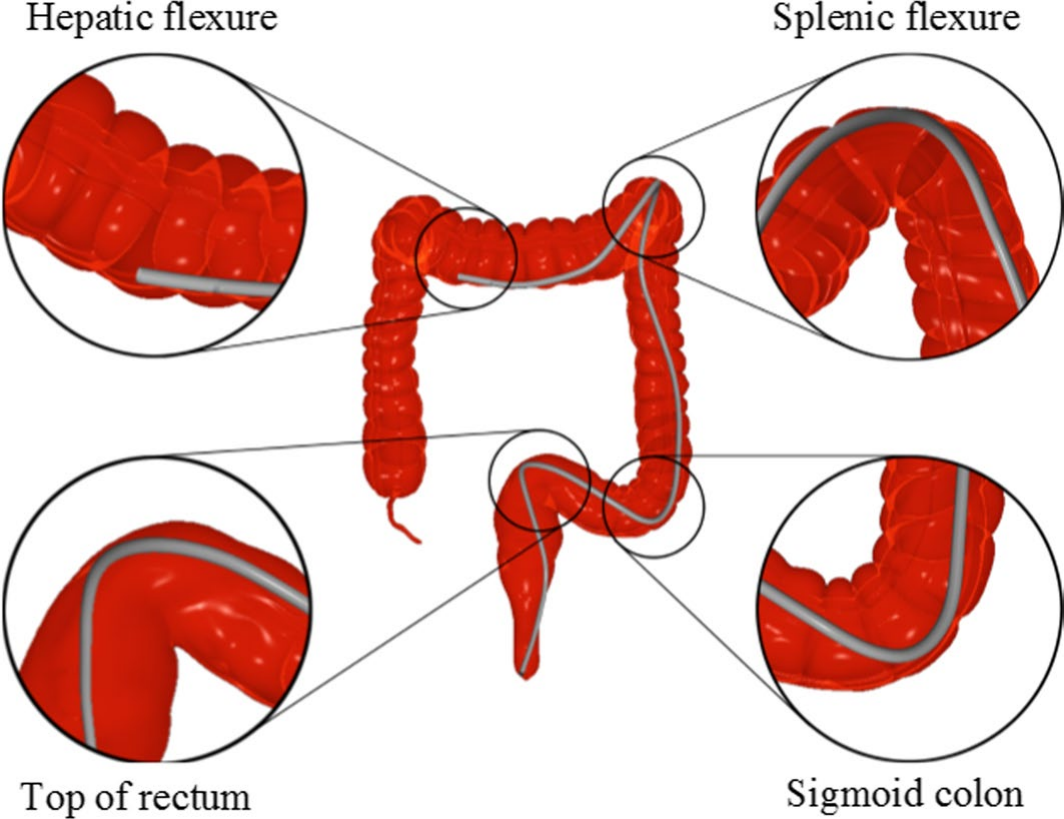
Graphical interface of simulator software component in virtual evironment

**Fig. 6:**
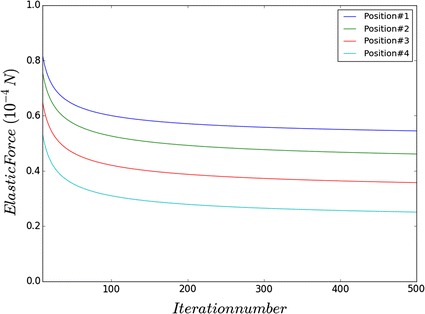
Variation of elastic force for the colonoscope in simulation position 1, 2, 3, and 4 that correspond to top of rectum, sigmoid colon, splenic flexure and hepatic flexure respectively

We use Microsoft Visual Studio 2010 as the main development environment. The Microsoft Foundation Class library and Windows API were applied to build the software component of the graphical user interface (GUI). Notably, C++ is used for the algorithms in subsequent sections, while the graphic programming is based on the OpenGL graphic library and GLSL shading language. The virtual simulation system we have developed is shown in Fig. 5 is equipped with Window 7 operating system, running at AMD AthlonTM II X2 processor at 3.00 GHz with a 4 GB system memory and a NVIDIA Quadro K2000 graphic card with 2 GB video memory. The main view in this system is endoscopic. The endoscopic view produces interior views of the colonic mucosa and distinguishing landmarks that the trainee must learn to identify and navigate to gain visuospatial awareness in colonoscopy. A significant example is the expectation of the trainee to identify ileocecal valve, appendiceal orifice, caecal folds, ileal mucosa etc. to confirm caecal intubation, or to navigate effectively through the transverse folds of the rectum. The endoscopic camera, along with haptic feedback, is the most valuable source of information for the trainee for maneuver the colonoscope effectively and to correctly identify any colonic polyps that may be candidates for biopsy/polypectomy (Fig. 6).

### Initial validation

To evaluate the simulation accuracy of the proposed physical model of instruments, validation experiments are carried out on a rectal-large bowel phantom which is made of transparent silicon tubing. Therefore, real colonoscopes used in clinical practice can be inserted into the rectal-bowel phantom. It is derived from a real human anatomy and thus provides a realistic environment for evaluating our simulated colonoscopy procedure. The bowel wall of the phantom has the flexibility equivalent to that implemented in our physical model. In our experiments, the phantom is first scanned by computed tomography with a spatial resolution of 0.75 mm and then a semi-automatic segmentation is applied on the 2D scanned images, followed by manual refinement by an expert physician. Finally, 3D surface geometry is obtained by the technique described in earlier papers.

We performed the same operations that navigate the colonoscope to the same position in both the phantom and the simulated model. We performed the same movements several time to guarantee that the simulated displacement of the colonoscope can be reproduced in the physical phantom model experiment. During this process, we took pictures to make comparisons between the simulated model and the phantom model.

Due to the refraction of the transparent silicon material, it is impossible to measure the actual distances of the colonoscope and the bowel walls. However, the relative distance, the shape of the colonoscope and the main collision points in pictures indicate that the behaviors of the virtual instruments in virtual models and the behaviours of the real ones in the real phantom are well matched visually.

### Parameter choosing

In experiment, we adopted different setting of η ranging from 2 to 15 times of the value of elastic coefficient to find a proper feedback coefficient. Then we carried out the 4 simulation positions with different setting of η and got similar results.

### User evaluation

The evaluation of our simulator was double-blinded tested by 15 experienced third-party gastroenterologists who were not involved in the design of our experiment. Questionnaire was designed to collect the feedback of the user’s experiences. The questionnaire has several evaluation criteria about the practical performance of the methods presented in this paper. Table 1 showed efficiency of our simulation can exactly meet the requirements of the physicians and our simulator can provide a realistic environment for core skills training in the colonoscopy procedures.

**Table 1:** The evaluation form 15 experienced third-party gastroenterologists

Evaluation criterion	Excellent	Very good	Good	Fair	Poor
Visual effect	10	3	2	0	0
Real-time interaction	15	0	0	0	0
Bend effect of colonoscope	3	5	7	0	0
Haptic experience of manipulation	9	6	0	0	0
Translation experience of colonoscope	12	3	0	0	0
Rotation experience of colonoscope	11	4	0	0	0
Behavior in complex colonic curvatures	10	5	0	0	0

### Time performance

Timing performance experiments with colonoscope represented by different numbers of nodes are carried out (Table 2). It is observed that with moderate discretization of virtual instruments the presented system can achieve real-time interactive visualization and high frame per second (FPS). For a scene with large amount of triangles, for example, the scene shown in Fig. 5 containing 623,805 triangles, our system still maintains nearly 60 FPS, which is suitable for an interactive simulation environment. The frame rate includes collision detection and response, endoscopic camera, colonoscope deformation and its visible light rendering.

**Table 2:** Time performance for the system including collision response and frame per second in different number of nodes

Number of nodes	Collision response (ms)	Frame per second
40	1.3	220
60	3.8	200
80	5.9	183
100	10.1	149
120	18.8	115
140	25.6	66

## Discussion

We present in this paper a high fidelity and fast colonoscopy simulator with the aim to train core skills of colonoscopy in a high yield yet safe environment. We also developed a series of strategies for accurate and high resolution anatomical representations, curve skeleton extraction and physics based instrument modelling. Various rendering technique are applied to ensure a real-time visualization by taking full advantage of the GPU computing capability.

The strengths of our system include good quality visual and haptic feedback as well as sophisticated haptic control and maneuvering of the virtual colonoscope inside realistic anatomical models. The trainee can interact with the virtual simulator with a real colonoscope and perform maneuvers such as pulling, pushing, applying torsion and translational motion to shorten the bowel and avoid high curvature formation.

Furthermore, more advanced techniques such as air/water insufflation and polypectomy can also be performed inside the simulation environment. Therefore, the trainee can acquire the necessary skills to identify and, using their clinical judgment, treat polyps found during the procedure.

Furthermore, we also plan to establish a set of performance parameters that can be used to objectively measure a trainee’s performance in colonoscopy. Such parameters include:

1. Time taken for caecal intubation;
2. Percentage of mucosa visualizer;
3. ‘Pain indicator’ or quantifiable measure of discomfort caused by colonoscope maneveurs;
4. Adenoma detection rate (ADR) (note: after consulting experienced endoscopists and reviewing publications we could potentially settle on a ‘mock ADR’). Then, using randomly generated cases, we could input adenomas into our generated cases proportionally according to the rate of our ‘mock ADR’.

When a trainee makes an error, the consequences will be reflected in the system. For example, if the bowel wall is perforated, then hemorrhage will occur and be simulated. By this way the trainee can see potential consequences on virtual patient and learn how to avoid and manage intraoperative catastrophic events.

This is a particularly notable advantage that our simulator will have over the traditional ‘see one, do one, teach one’ model. It is extremely difficult if not impossible to ensure that trainees experience a full range of complications because they are not common in real life clinical situations. Therefore, modelling complications such as haemorrhage or perforation could become crucial aspects of our simulator, as the trainee will then be able to learn to manage serious complications without any harm occurring patients. The colon can be modelled using discrete elements to give more flexibility in future studies [24–26].

Furthermore, the current bowel patient database will be updated to include more challenging cases including sharper angulations and thinner colon walls. These bowel characteristics will test the more advanced trainee, ensuring that the simulator does not become boring or stale after multiple use.

**Fig. 7:**
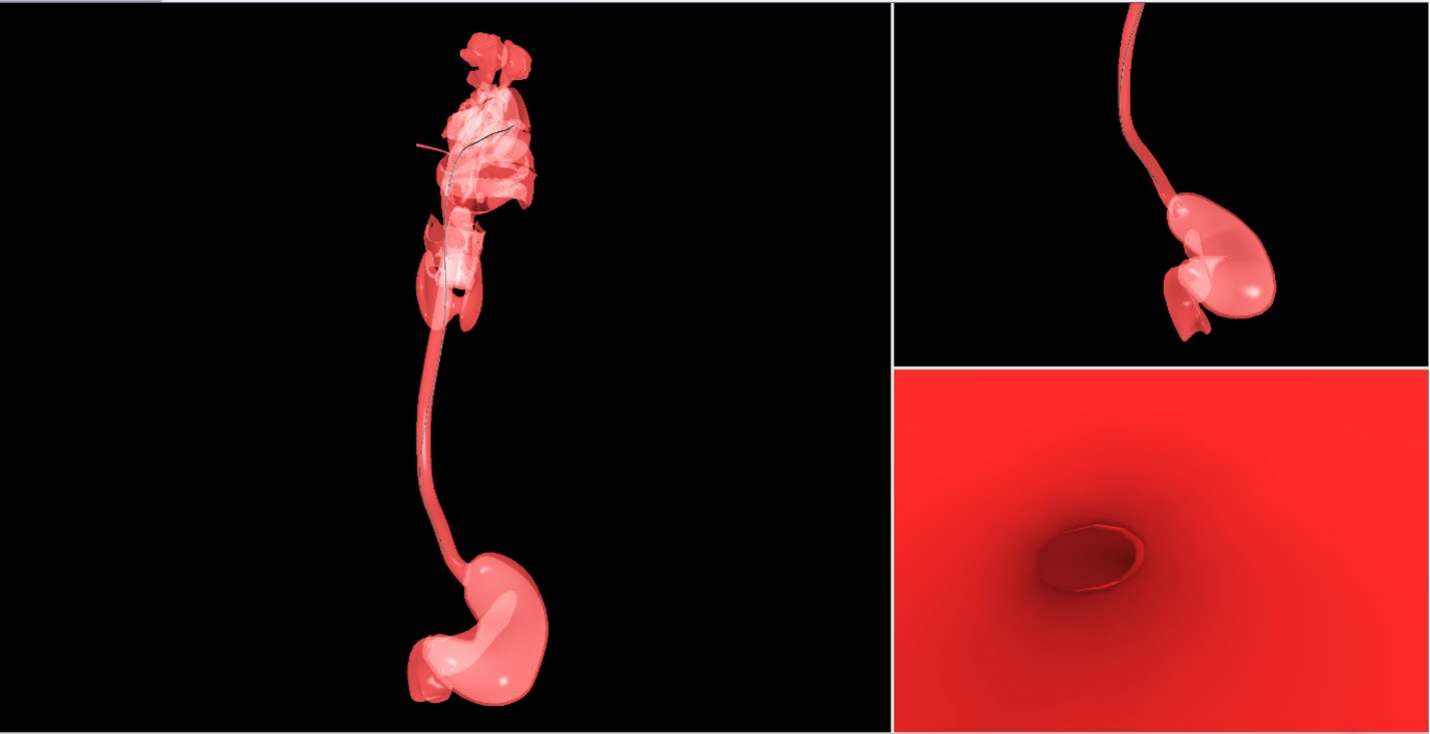
The catheter can be seen intubated into the nasal aperture with the nasopharyngeal–oesophageal easily passage visualised

In addition we have tried to utilize this approach for procedure of high-resolution oesophageal manometry (HRM) that is an emergent technique to measure oesophageal motility. It has evolved from the conventional manometry to measure pressures at every centimetre of the oesophagus. We developed a HRM simulator to help the trainee gain a visuospatial awareness of the location of catheter while advancing it. This visual feedback (see also Fig. 7, coupled with the haptic feedback from our device (figure with haptic controller here), allows the trainee to become familiar with maneuvering the HRM catheter.

## Conclusion

Currently, our prototype has been validated in terms of the physical modeling of instruments. However, upon the completion of several additional components that are ongoing development, it will undergo face and content validity from our hospital collaborators and then a comprehensive assessment and validation study including concurrent and predictive validity.

As previously alluded to, the ultimate utility of our simulator is not limited to providing trainees in a highly immersive and realistic environment. We also hope to provide more experienced endoscopists with a predictive tool in which patient-specific data can be inputted in order to plan a high risk or difficult colonoscopy procedure.
